# Massive benign pericardial cyst presenting with simultaneous superior vena cava and middle lobe syndromes

**DOI:** 10.1186/1749-8090-3-32

**Published:** 2008-05-21

**Authors:** Pankaj Kaul, Kalyana Javangula, Shahme A Farook

**Affiliations:** 1 Yorkshire Heart Centre, Leeds General Infirmary, Great George Street, Leeds, LS1 3EX, UK

## Abstract

A 66 year old woman presented in extremis with symptoms and clinical and radiological signs of simultaneous obstruction of superior vena cava and middle lobe of right lung secondary to compression by a massive benign anterior mediastinal cyst. Excision of the cyst at median sternotomy resulted in complete resolution of all symptoms. This report is unusual on account of a) the concomitant presence of superior vena cava and middle lobe syndromes caused by a benign cyst because of its sheer size producing obstruction of these structures and b) the complete resolution of all symptoms and signs after removal of the cyst. Benign anterior mediastinal cysts are unknown to cause either of the two syndromes. To our knowledge, it is the first report of a benign anterior mediastinal cyst causing either superior vena cava syndrome or middle lobe syndrome or both simultaneously. Etiologies of both superior vena cava and middle lobe syndromes are discussed in detail.

## Case presentation

**Figure 1:**
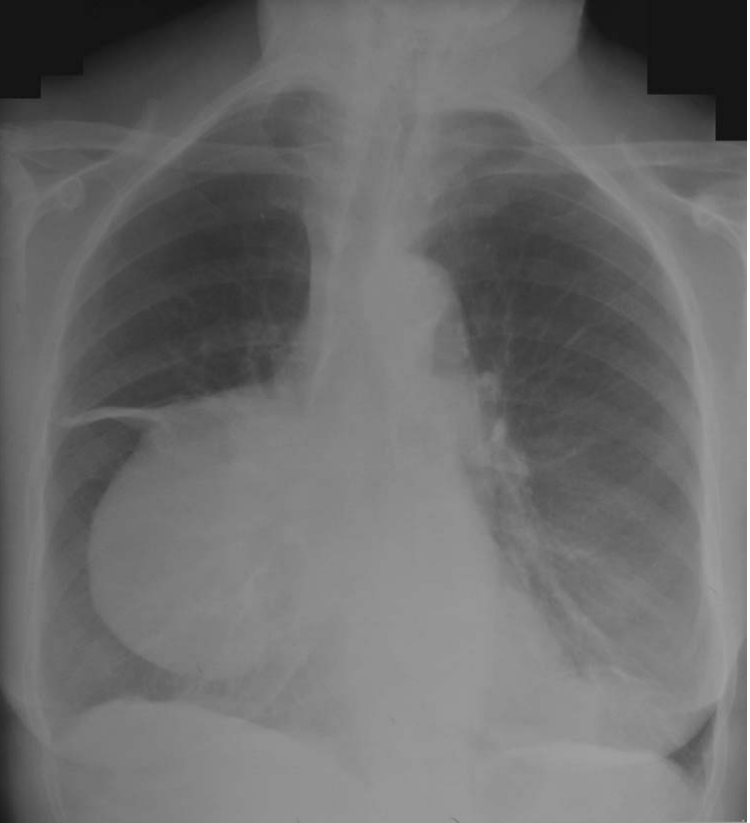
Chest X ray (PA) view showing a large mediastinal mass occupying middle and lower zones of right chest and causing compressive atelectasis of middle lobe.

**Figure 2:**
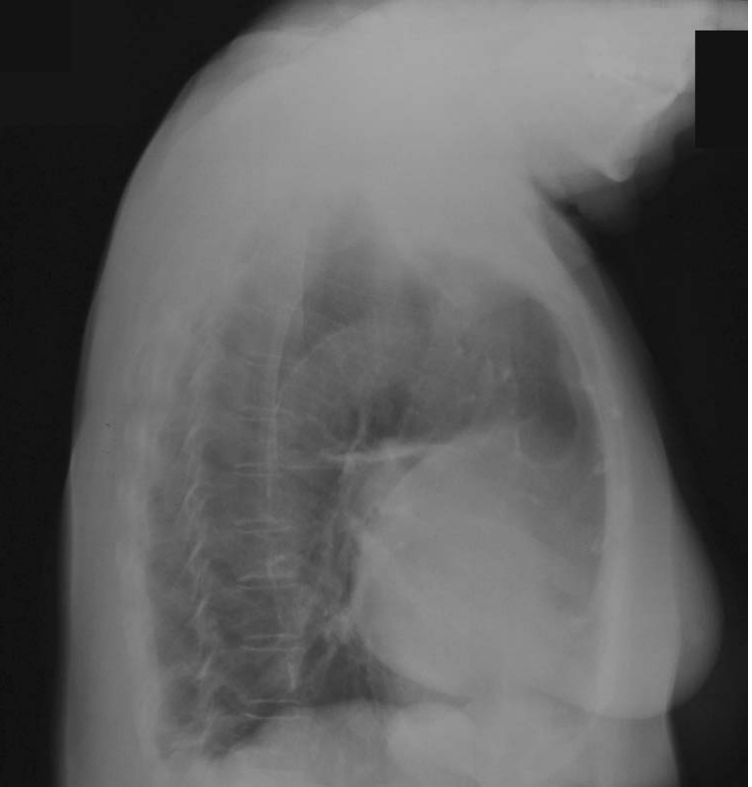
Chest X ray (right lateral view) confirms the anterior location of the mediastinal mass.

**Figure 3:**
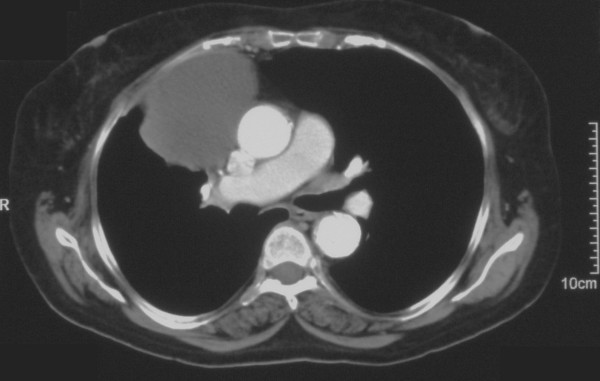
CT scan of chest showing a smooth ovoid low density mass abutting the right anterior chest wall, diaphragm and the right atrium and ventricle.

**Figure 4:**
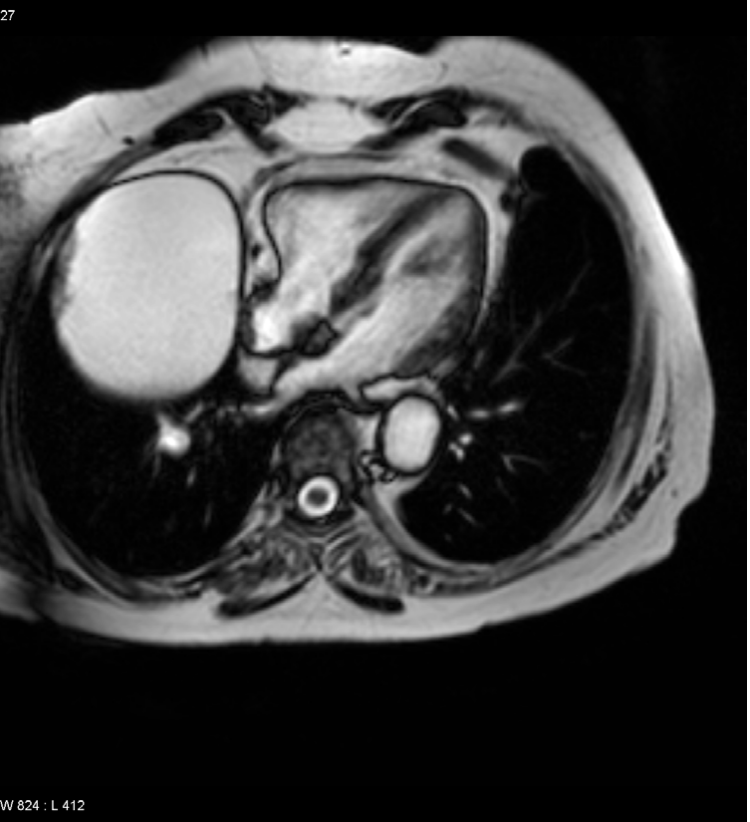
MR scan demonstrating a large cystic mass in right anterior hemithorax.

**Figure 5:**
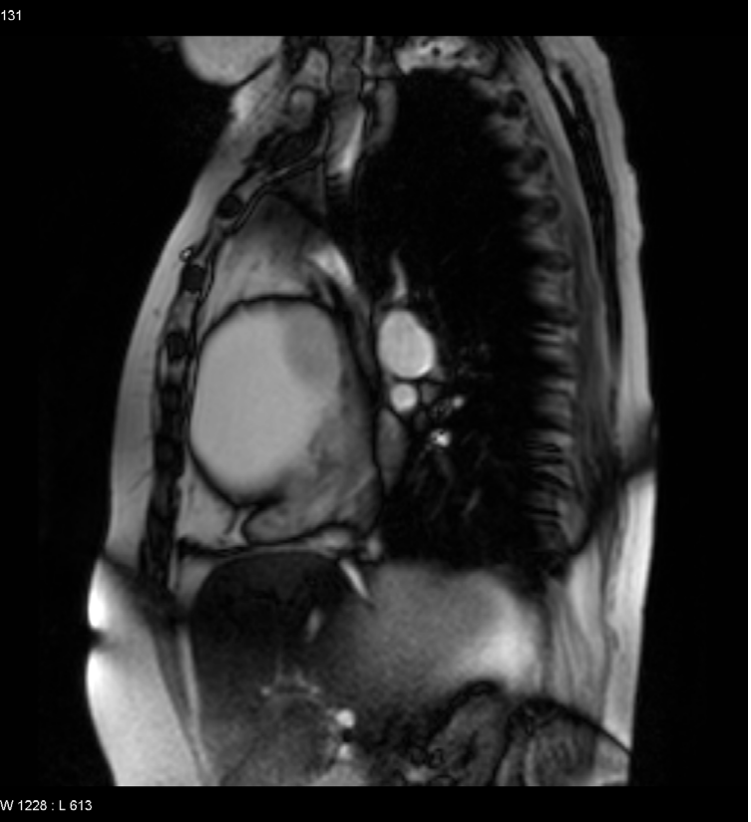
MR scan showing external compression of SVC, right atrium and right ventricle.

**Figure 6:**
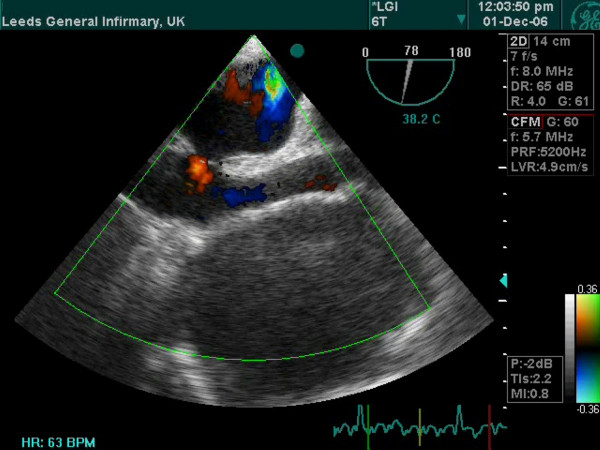
Transesophageal echocardiogram shows a huge anterior mediastinal fluid filled mass.

A 66 year old hypertensive and asthmatic chronic smoker presented with 8 month history of progressively increasing shortness of breath. Examination revealed an anxious, tachycardic woman, breathless at rest with engorged neck veins, purple discolouration of face, swelling of face and neck and wheeze over whole of right chest. A posteroanterior chest X ray showed a large mediastinal mass occupying right middle and lower zones of chest with an atelectatic middle lobe (fig 1). Lateral chest x ray confirmed the anterior location of the mediastinal mass (fig 2). Spirometry demonstrated FEV1 0.84 L (47% predicted), FVC 2 L (92% predicted), VC 2 L (92% predicted), FEV1/FVC 42%, PEF120 L/min. Flexible bronchoscopy showed normal appearances of the tracheobronchial tree. CT Thorax showed a smooth ovoid mass in the right anterior lower chest abutting the chest wall, diaphragm and the right pericardium, and which showed a thin slightly higher density wall and low density contents with average CT number of 10, consistent with fluid (fig 3). There was no mediastinal lymphadenopathy. An MR scan showed a large cystic mass 11 × 11 × 8 cm in the right anterior hemithorax, having the signal characteristics of neither a vascular lesion nor a lipoma, in direct contact with pericardium and, therefore, quite likely to be a pericardial cyst, and causing external compression of right hilum, right atrium and SVC (figs 4 and 5). Blood examination revealed normal FBC, U&E, LFTs, calcium and glucose and a slightly increased ESR at 25 mm/hr. Echocardiography revealed an extracardiac mass abutting the right atrium and ventricle and TOE, on operation table, confirmed the presence of a huge anterior mediastinal mass (Fig 6).

**Figure 7:**
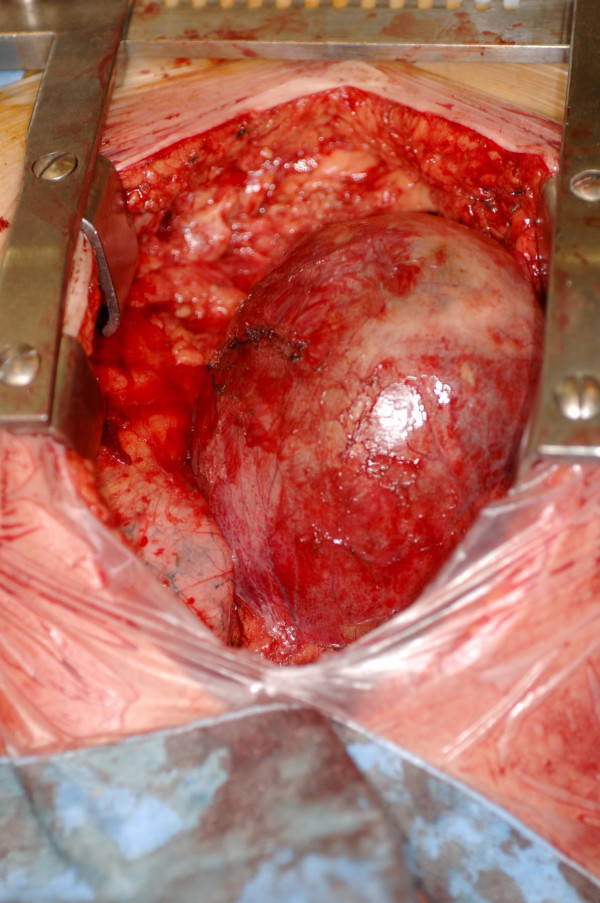
Intraoperative photograph shows the cyst filling up almost the entire anterior mediastinum, overlying SVC, right atrium, right pulmonary hilum, the middle lobe and the medial basal segment of the lower lobe.

**Figure 8:**
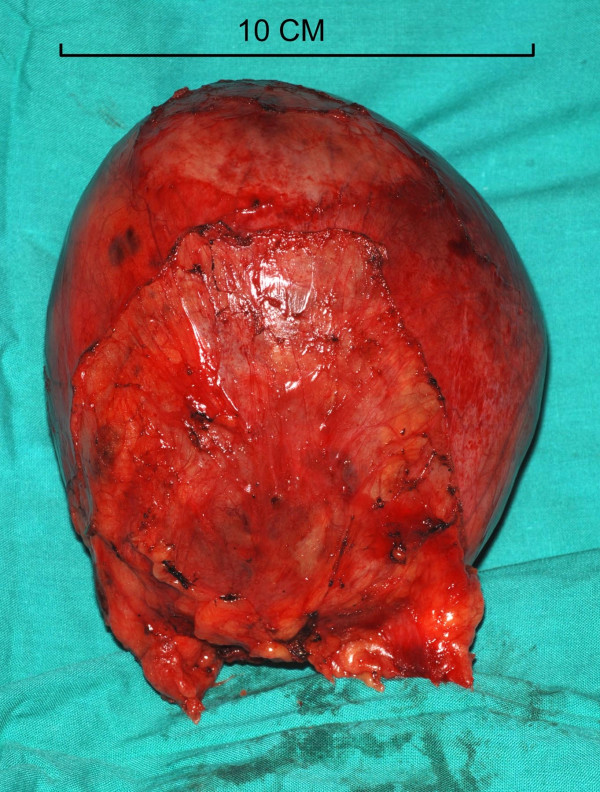
Intraoperative photograph of the intact smooth walled cyst.

**Figure 9:**
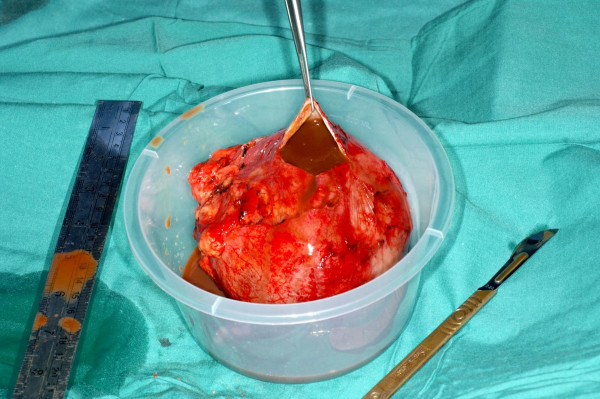
Intraoperative photograph showing the thin walled cyst filled with haemorrhagic fluid.

**Figure 10:**
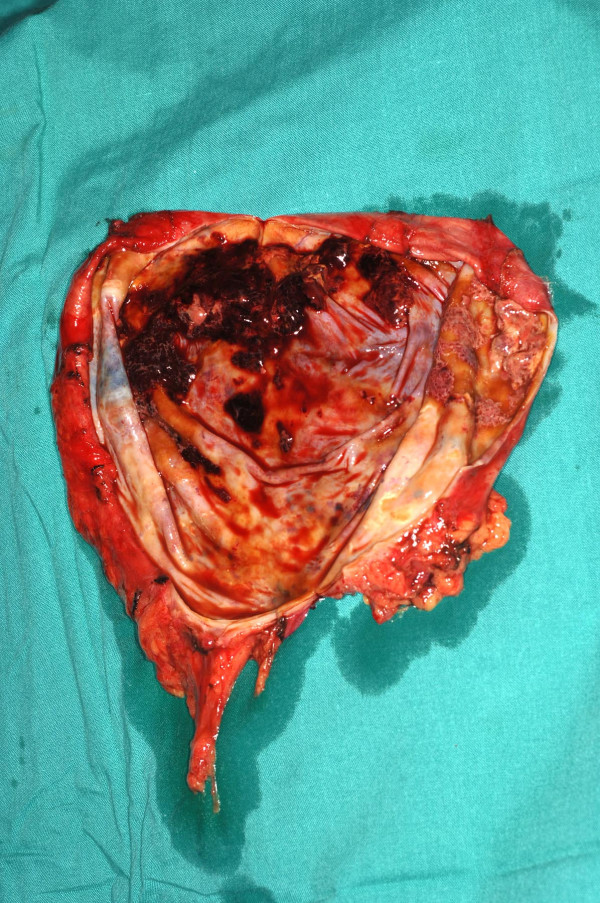
Intraoperative photograph showing only a few clots and no suspicious masses.

**Figure 11:**
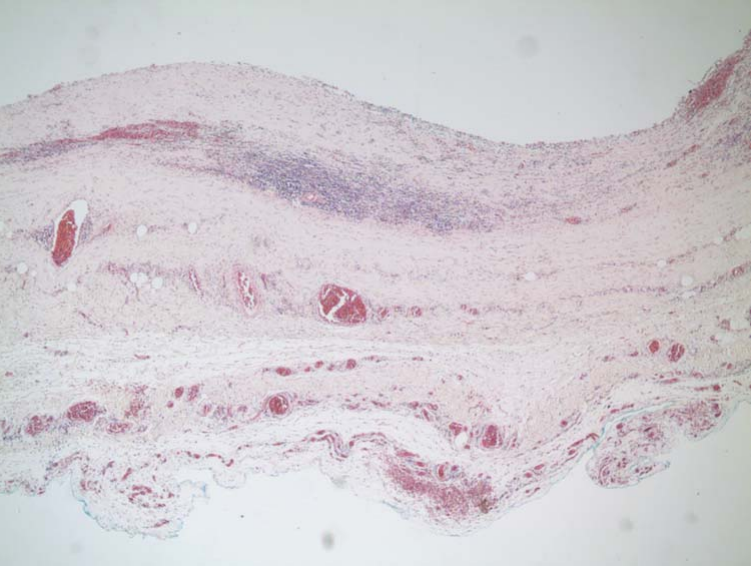
Histopathology shows a fibrous walled cyst with no lining  epithelium with chronic inflammatory cells and hemosiderin.

**Figure 12:**
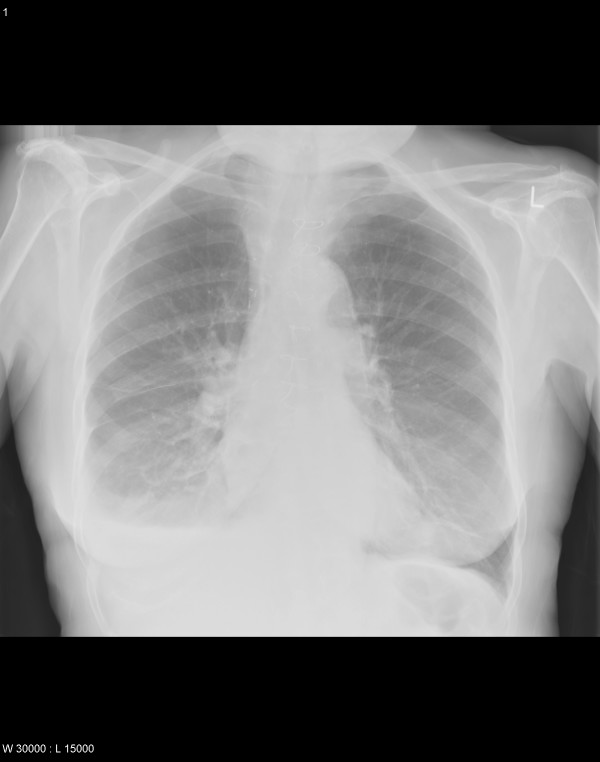
Postoperative chest X ray (PA view) showing complete expansion of middle lobe.

At median sternotomy, there was a 15 × 10 × 8 cm cyst, adherent to the pericardium loosely, overlying SVC, right atrium, right pulmonary hilum, the middle lobe and the anterior basal segment of the lower lobe of lung and compressing all the above structures (Fig 7). The large cyst was dissected off intact from the above structures without opening the pericardium while preserving the right phrenic nerve (Fig 8). The middle lobe and the anterior basal segment of lower lobe expanded completely. The cyst was opened on table (Fig 9). It had a thin 2 mm wall, was filled with 600 mls of haemorrhagic fluid with strands of fibrin and the inner wall did not have any suspicious masses although there were a few small clots attached to it (Fig 10). Histopathology revealed a fibrous-walled cyst with no lining epithelium. Within the fibrous wall, chronic inflammatory cells and haemosiderin pigment were noted. No malignancy was noted (fig 11). Patient made uncomplicated recovery. Her facial swelling, rubescent discolouration and venous distension resolved, tachycardia disappeared and shortness of breath improved dramatically. Postoperative chest X ray showed near complete expansion of right lung (fig 12) and she was discharged home 7 days after her operation. At follow up, 6 weeks later, she was completely asymptomatic.

## Discussion

Primary mediastinal cysts constitute approximately one fifth of all mediastinal masses. The cysts may originate from pleura or pericardium, tracheobronchial tree, gastrointestinal tract, neurogenic tissue, thymus gland or lymphoid tissue. Benign teratomas may present as epidermoid cysts, dermoid cysts or cystic teratomas [1]. Mediastinal cystic masses may also result from specific or non-specific infections or parasitic infestations like Echinococcus [2].

Anterior mediastinal cysts most commonly are pleuropericardial, thymic, teratomatous or cystic hygromas.

Pleuropericardial cysts are benign mesothelial cysts that arise as a result of persistence of one of the mesenchymal lacunae that normally fuse to form the pericardial sac [3], or, as suggested by Lillie [4], due to the failure of an embryological ventral diverticulum to fuse. Alternatively, they may be believed to arise from the infolding of the advancing edge of the pleura during its embryological development. These cysts are unilocular, contain clear watery fluid, present typically in anterior cardiophrenic angle, more often on right side than left. Microscopically, the wall has a single layer of mesothelial cells resting on a loose stroma of connective tissue.

True thymic cysts are thin walled, unilocular and contain normal thymic tissue within their walls and arise from third branchial pouch. Microscopically, the wall is lined by low cuboidal epithelium. However, malignant degeneration within a thymoma may result in a cystic thymoma, with a residual mass projecting into the cavity of the cyst from the wall.

Typically, lymphangiomas arise from neck and extend into mediastinum. They contain chyle and are classified according to the size of the spaces into cystic hygromas or cavernous lymphangiomas. Cystic hygromas are multiloculated, and a mediastinal hygroma is almost always an extension of a cervical hygroma. However, rarely, a uniloculated primary anterior mediastinal lymphogenous cyst containing yellow or brown fluid may be found [5].

Teratodermoids are classified generically as benign germ cell tumours. They are further divided into three categories: epidermoid cysts which are lined by simple squamous cell epithelium, dermoid cysts which have squamous epithelial lining containing elements of skin appendages like hair and sebaceous glands and teratomas which may be solid or cystic and contain identifiable cellular elements of two or three germinal layers [1].

Our patient had a massive fibrous but thin walled cyst filled with brownish fluid. There was no lining epithelium but there was chronic inflammatory cell infiltration with lymphocytes, plasma cells, histiocytes, giant cells containing pigment and lymphoid aggregates seen with no evidence of malignancy. Considering the location of the cyst in the anterior mediastinum and right hemithorax, it is likely that this was originally a pleuropericardial or thymic cyst whose epithelial lining was destroyed by inflammation.

Benign anterior mediastinal cysts of pleuropericardial or thymic origin in adult are mostly asymptomatic unless they get secondarily infected which is unusual. Bronchogenic cysts, which almost always arise in the visceral compartment, in close relation to the trachea or bronchi, get infected more frequently, particularly when there is a persistent communication with the tracheobronchial tree. It is extremely unusual for benign anterior mediastinal cysts to cause compressive mediastinal symptoms, because most normal, mobile mediastinal structures can conform to pressure. Besides, anterior mediastinal cysts enlarge anteriorly and inferiorly, in the path of least resistance.

Extrinsic or intrinsic compression of superior vena cava (SVC) from a variety of benign and malignant processes results in congestion of venous outflow from head, neck and upper extremity. Important factors that determine the nature of presentation include the rate and completeness of obstruction, the etiology of obstruction and its site relative to the drainage of azygous vein. Although syphilitic aneurysms were responsible for nearly half of all reported cases in the first half of the twentieth century [6], the most common cause, in the current era, is malignancy, with lung cancer being the most frequent cancer, the small cell lung cancer being the most frequent cell type [7] and lymphoma being the second most frequent tumor. Less than 5% of superior vena cava obstruction is caused by benign disease, although the exponential increase in the number of cardiac catheterizations, indwelling central venous catheters, whether for prolonged monitoring by pulmonary artery Swan-Ganz catheters or for long term antibiotic or cancer chemotherapy with Hickman lines, as well as transvenous pacemaker leads shall surely lead to a revision of this conservative estimate. The other benign causes of superior vena cava obstruction include substernal goitre, Riedel's thyroiditis, fibrosing mediastinitis, and aneurysms of brachiocephalic vessels. Postoperatively, SVC obstruction can follow cardiac transplantation, Mustard operation for transposition of great vessels, and, rarely, rechannelling of partial anomalous pulmonary venous drainage.

An atelectatic middle lobe caused by extrinsic bronchial compression of the long, slender and vulnerable middle lobe bronchus, by malignant or inflammatory lymph nodes, whether demonstrated or not, has long been called middle lobe syndrome. Admittedly, the middle lobe has a poorer drainage than the other lobes, is comprehensively surrounded by lymph nodes and has very little collateral ventilation. However, middle lobe syndrome can be caused by entities as diverse as carcinoma of middle lobe bronchus, mucosa associated lymphoid tissue (MALT) lymphomas [8] or sclerosing Hodgkin's disease, primary benign endobronchial diseases like tuberculosis [9] and sarcoidosis, strictures of unknown etiology, foreign bodies[10], fibrosing or resolving pneumonitis, sclerosing mediastinitis, histoplasmosis, aspergillus [11] or blastomyces [12] infections, obliterative bronchitis [13], bronchiectasis, bronchial hyper-responsiveness (BHR) and asthma [14] and, rarely, bronchogenic or esophageal duplication cysts.

Our patient presented with simultaneous superior vena cava and middle lobe syndromes as well as compressive atelectasis of medial basal segment of the lower lobe of the right lung. Even though benign anterior mediastinal cysts are not known to cause either of the two syndromes, it was the sheer size and the weight of the cyst that was responsible for causing such significant obstruction of both of these vital anatomic structures. Removal of the cyst resulted in instantaneous relief of both obstruction of superior venecava and the compressive atelectasis of middle lobe and medial basal segments of the right lower lobe.

## Competing interests

The authors declare that they have no competing interests.

## Authors' contributions

PK conceived, designed and drafted the manuscript, KJ participated in the design of the study and helped in the collection of figures, SAF carried out the preliminary studies. All authors read and approved the manuscript

## Note

We would like to acknowledge the help of the following at Leeds General Infirmary: Dr Ramnath, Consultant Pathologist with the histopathology slides, Mr Stuart Powell, Medical Illustration Unit and Dr S. Balaji, Consultant Anaesthetist, with echocardiographic images.

Written informed consent was obtained from the patient for publication of this case report and accompanying images. A copy of the written consent is available for review by Editor-in-Chief of this journal.
